# Small RNAs for defence and regulation in archaea

**DOI:** 10.1007/s00792-012-0469-5

**Published:** 2012-07-05

**Authors:** Anita Marchfelder, Susan Fischer, Jutta Brendel, Britta Stoll, Lisa-Katharina Maier, Dominik Jäger, Daniela Prasse, André Plagens, Ruth A. Schmitz, Lennart Randau

**Affiliations:** 1 Biologie II, Ulm University, Albert-Einstein-Allee 11, 89069 Ulm, Germany; 2 Institute for General Microbiology, Christian-Albrechts-University Kiel, Am Botanischen Garten 1-9, 24118 Kiel, Germany; 3 Prokaryotic Small RNA Biology, Max Planck Institute for Terrestrial Microbiology, Karl-von-Frisch Strasse 10, 35043 Marburg, Germany

**Keywords:** sRNA, Lsm, Hfq, Archaea, CRISPR, crRNA

## Abstract

Non-coding RNAs are key players in many cellular processes within organisms from all three domains of life. The range and diversity of small RNA functions beyond their involvement in translation and RNA processing was first recognized for eukaryotes and bacteria. Since then, small RNAs were also found to be abundant in archaea. Their functions include the regulation of gene expression and the establishment of immunity against invading mobile genetic elements. This review summarizes our current knowledge about small RNAs used for regulation and defence in archaea.

## Small RNAs regulate gene expression

Bacteria and eukaryotes use a plethora of non-coding RNAs to regulate gene expression (Hüttenhofer et al. 2005; Brantl 2009; Waters and Storz 2009; Krol 2010). The mechanisms by which bacterial as well as eukaryotic small RNAs act have been studied in detail revealing common characteristics as well as differences. Bacterial small regulatory RNAs are often required for regulation of metabolic pathways (Gottesman 2004a, b). Bacterial *trans*-encoded sRNAs can act by masking the ribosome binding site or the start codon or binding to sequences close to these sites (Gottesman 2004a, b; Waters and Storz 2009). *Trans*-encoded sRNAs are only partially complementary to their target and often require the help of a protein (i.e. Hfq) for activity. In the last years more and more bacterial RNA populations have been analyzed with deep-sequencing methodologies revealing also a high amount of *cis*-encoded antisense RNAs (Rasmussen et al. 2009; Thomason and Storz 2010; Georg and Hess 2011; Brantl 2012). *Cis*-antisense RNAs are encoded on the opposite strand of a gene and are therefore completely complementary to their target. It is estimated that a bacterial genome encodes about 200–300 sRNAs and in *Escherichia coli* for instance about 140 sRNAs are known but a biological role has been defined for only 25 of these, showing how difficult it is to unravel their in vivo function (Brantl 2012). In addition, sRNAs like the 6S RNA (Gildehaus et al. 2007) have been shown to regulate gene expression by specific binding to proteins (Brantl 2009).

In eukaryotes, the majority of the non-coding miRNAs binds to the 3′ end of the target mRNAs, which triggers degradation or leads to inhibition of translation (Meister 2007; Guo et al. 2010; Krol 2010). It has been predicted that 30–50 % of all human genes are regulated by miRNAs, emphasizing the importance of small RNAs in regulation of gene expression (Lewis et al. 2005; Krol 2010).

It has been suggested that archaea also use small RNAs to regulate gene expression, but at the moment we do not know details about the molecular mechanism involved in archaeal sRNA regulation. Up to date in only six archaeal organisms the small RNA population has been investigated: *Archaeoglobus fulgidus*, *Sulfolobus solfataricus*, *Methanocaldococcus jannaschii*, *Methanosarcina mazei*, *Pyrococcus furiosus*, and *Haloferax volcanii* (Klein et al. 2002; Schattner 2002; Tang et al. 2002, 2005; Zago et al. 2005; Jäger et al. 2009; Soppa et al. 2009; Straub et al. 2009; Wurtzel et al. 2009; Fischer et al. 2010; Babski et al. 2011). Details about the site and the mode of the interactions between the sRNA and the target are not known and it is therefore not clear whether additional factors are required.

Here, we present the current state of the art about sRNAs in Archaea, their diversity and potential biological functions.

## Elucidation and analysis of the archaeal sRNA population

The first approaches to identify small RNA populations in archaeal organisms were carried out 10 years ago using experimental and computational methods.

### Prediction of sRNA genes

The prediction of non-coding RNA genes is not as straightforward as the prediction of protein coding genes. In bacterial organisms where promoter and terminator elements have been clearly defined those can be used as a tool to predict sRNA genes. This approach has been used successfully together with comparative genome analysis to predict sRNAs in *E. coli* (Argaman et al. 2001; Wassarman et al. 2001). Although promoter and terminator elements have also been defined for archaeal genes, in silico prediction of sRNA genes using these parameters was not successful to date (Soppa et al. 2009; Jäger, Schmitz, Liesegang, unpublished). In addition, for only 38 % of the identified sRNA genes from *Haloferax* and 44 % of the sRNA genes from *Methanosarcina* basal promoter elements were found in an appropriate distance to the transcriptional start site, suggesting that either the sRNAs are processed from precursors or that the sRNA genes have unusual promoter elements. Therefore, other approaches for sRNA prediction are used one being the analysis of the GC content. For example, in hyperthermophiles non-coding RNAs have a higher GC content and thus non-coding RNAs are predicted by the identification of GC rich regions. Another approach employed for sRNA gene prediction is comparative genome analysis, here non-coding RNAs are identified as intergenic regions conserved between at least two organisms.

The first bioinformatics approach to identify archaeal small RNAs was applied in *M. jannaschii* and *P. furiosus* (Klein et al. 2002; Schattner 2002) (Table 1). Since both organisms have a high A/T content, the screen for novel small RNAs used a GC content bias as well as the programme QRNA finder, which uses a comparative sequence analysis algorithm to detect conserved structural RNAs (Rivas and Eddy 2001). Using this method five new small RNAs were identified in both organisms. The second approach used local base composition statistics to identify small RNAs in *M. jannaschii*. This search resulted in the identification of 18 putative new small RNAs (Schattner 2002). It took another couple of years until sRNA predictions for a third archaeon, *H. volcanii*, were published. Here, two separate bioinformatics approaches were used to elucidate the small RNA population (Babski et al. 2011). Comparison of intergenic regions of *H. volcanii* with one halophilic bacterium, one crenarchaeal species and three haloarchaeal species was used in the first approach. Since genomes from different phylogenetic groups were used for comparison only highly conserved sRNAs could be identified, resulting in the prediction of 31 sRNAs. A comparative analysis restricted to haloarchaeal organisms was used in the second bioinformatics approach; here, 94 putative sRNA genes were identified, which were conserved in at least two or three haloarchaea.

**Table 1:** sRNAs identified in Archaea

Type of sRNA	*M. janaschii*		*P. fu*	*H. volcanii*			
(a) In silico identification							
Prediction	(1)	(2)		(1)	(2)		
Intergenic sRNA	5	18	5	31	94		

Type of sRNA	*A. fu*	*S. solfataricus*			*H. volcanii*		*M. ma*
Method of identification	RNomics	RNomics	CoIP	HTS	RNomics	HTS	HTS
(b) Experimental identification							
Intergenic sRNA	9	11	3	125	21	145	199
*cis*-Antisense	33	19	8	185	18	45	43

(a) In silico approaches were used to predict sRNAs in *M. jannaschii* (Klein et al. 2002; Schattner 2002); *P. furiosus* (P. fu) (Klein et al. 2002) and *H. volcanii* (Babski et al. 2011). The number of sRNAs identified with these approaches is shown. For *M. jannaschii* and *H. volcanii* two different approaches were used

(b) The sRNA populations from *A. fulgidus* (A.fu) (Tang et al. 2002); *S. solfataricus* (Tang et al. 2002; Hüttenhofer et al. 2005; Zago et al. 2005; Wurtzel et al. 2009), *H. volcanii* (Straub et al. 2009; Heyer et al. 2012) and *M. mazei* (M. ma) (Jäger et al. 2009). The number of sRNAs identified with experimental RNomics, co-immuno precipitation (CoIP) or HTS is shown

The prediction of sRNAs is an important tool for the analysis of regulatory RNAs but for the predicted sRNA candidates an experimental verification is essential, since the identified conserved structures might be conserved riboswitch-like regulatory elements which are part of a 5′ or 3′ UTR and do not represent sRNA genes.

### Experimental identification of small RNAs

The first experimental identification of an archaeal sRNA population was performed with the euryarchaeon *Archaeoglobus fulgidus* (Tang et al. 2002). Tang et al. generated a cDNA library from a size selected RNA fraction (50–500 nt). Sequencing revealed nine potential *trans*-encoded sRNAs and 33 *cis*-antisense sRNAs, in addition 22 short RNA involved in the CRISPR/Cas prokaryotic immune system (crRNAs) were found.

The next set of archaeal sRNAs was analyzed in the crenarchaeon *Sulfolobus solfataricus.* Here, two different approaches were used: experimental RNomics (Tang et al. 2005) and co-immuno-precipitation with the protein L7Ae (Zago et al. 2005). Later a high through sequencing approach was also applied (Wurtzel et al. 2009). In the experimental RNomics approach a cDNA library representing the RNA population from 50 to 500 nucleotides was generated. To avoid identification of low level unspecific transcripts novel small RNA candidates were only further investigated if their expression was confirmed by northern analyses. This approach identified 19 *cis*-antisense sRNAs and 11 *trans*-encoded sRNAs and one crRNA. The majority of the *cis*-antisense sRNAs was encoded opposite to transposase genes, suggesting that the sRNAs are involved in regulation of transposons (Tang et al. 2005). For the co-immunoprecipitation approach, the *Sulfolobus* L7Ae protein was used to isolate sRNAs (Zago et al. 2005). The archaeal L7Ae protein is a component of the large subunit of the ribosome and part of ribonucleoprotein complexes (RNPs) which are responsible for ribose methylation and pseudouridylation (Zago et al. 2005). The L7Ae protein has been shown to bind to archaeal snoRNAs (Kuhn et al. 2002; Rozhdestvensky et al. 2003). The co-immuno-precipitation with L7Ae resulted in the identification of three *trans* sRNAs and five *cis*-antisense RNAs (Zago et al. 2005). The HTS approach detected 310 sRNAs including all formerly identified sRNAs (Wurtzel et al. 2009). Interestingly, more *cis*-antisense sRNAs (185) than *trans*-encoded sRNA (125) were detected. Although an increasing number of *cis*-antisense sRNAs are being identified, the number of *trans*-encoded sRNAs has so far been higher than the number of *cis*-antisense sRNAs found. This is the first case, where *cis*-antisense RNAs outnumber the *trans*-encoded sRNAs (Wurtzel et al. 2009).

In the same year the sRNA populations of two euryarchaeota were published: *M. mazei* and *H. volcanii*. RNAseq analysis of *M. mazei* growing under nitrogen starvation and nitrogen saturation identified 199 *trans*-encoded sRNAs as well as 43 *cis*-antisense sRNAs in this organism (Jäger et al. 2009). Comparative genome analysis further revealed that a significant number of the identified *cis*-antisense sRNAs (30 %) and the *trans*-encoded sRNAs (21 %) were conserved in *M. mazei* and *Methanosarcina* *bakeri* and *M.* *acetivorans*. Several of the *cis*-antisense sRNA candidates are encoded on the opposite strand to transposase genes indicating a post-transcriptional regulation of transposon mobility by antisense RNAs, a mechanism previously observed for insertion elements in *S. solfataricus* (Tang et al. 2005) and the bacterial transposons Tn*10* and Tn*30* in *Escherichia coli* (Ma and Simons 1990; Arini et al. 1997). The finding that in *M. mazei* several of those *cis*-antisense sRNAs show different expression levels depending on the nitrogen source (Jäger et al. 2009), strongly suggests that transposition events in *M. mazei* are regulated in response to the nitrogen availability.

40 *trans*-encoded sRNA candidates have the potential to encode peptides smaller than 30 amino acids. As the majority of those small potential ORFs as well as the flanking non-coding RNA region show high conservation in *Methanosarcina* *bakeri* and *M.* *acetivorans* it is tempting to speculate that those *trans*-encoded sRNAs might have a dual function as regulatory sRNA and mRNA. Very recently three of those small peptides have been shown to be expressed (Prasse, Jäger, Thomsen, Becher, Hecker and Schmitz, unpublished). Up to now the expression of 88 out of 130 randomly selected sRNA candidates was confirmed using northern blot analyses. Differential expression in response to the nitrogen source was confirmed for 18 small RNAs, representing the first prokaryotic regulatory RNAs potentially involved in nitrogen stress response. Based on these findings and the strong conservation a crucial role of sRNAs in the nitrogen or general stress response has been proposed for *M. mazei* (Jäger et al. 2009).

For the identification of sRNAs in the halophilic archaeon *H. volcanii* three different experimental approaches were employed, i.e. experimental RNomics (Straub et al. 2009), HTS (Heyer et al. 2012) and co-purification with the Lsm protein (Fischer et al. 2010). Altogether 145 intergenic sRNAs were identified and 45 *cis*-antisense RNAs (Table 1).

For the experimental RNomics approach, a cDNA library was generated from a *Haloferax* RNA fraction containing RNA molecules ranging in size from 130 to 460 nts. Sequencing of this library lead to the identification of 18 antisense sRNAs and 21 intergenic sRNAs (Straub et al. 2009). Northern analyses of the candidate sRNAs showed differential expression of several sRNAs. One sRNA has the potential to code for a peptide of 34 amino acids length, which is conserved between haloarchaeal organisms.

In a second approach, a tagged version of the *Haloferax* Lsm protein was used to co-immuno-precipitate sRNAs (Fischer et al. 2010). The archaeal Lsm protein is like the bacterial Hfq protein a member of the Sm/Lsm protein family. Proteins of this family have been shown to be important players in cellular RNA pathways and in many bacteria the Hfq protein is required for interaction of some *trans*-encoded sRNAs with their target mRNAs. Expression of a FLAG-tagged Lsm protein in *Haloferax* cells confirmed that the archaeal protein binds to small RNAs. Sequencing of the co-immuno-precipitated RNAs revealed that 10 intergenic sRNAs and 7 of the bioinformatically predicted sRNAs were bound to the Lsm protein (Fischer et al. 2010). These data suggest that in archaea the Lsm protein might be involved in the sRNA regulation pathway.

In the third experimental approach cDNA libraries generated from size selected RNAs (17–500 nts) were sequenced using HTS. Six different cDNA libraries were constructed from RNA isolated from cultures grown at standard growth conditions, low salt conditions and high temperature, each at exponential and stationary phase. Altogether 145 *trans*-encoded sRNAs and 45 *cis*-antisense RNAs were identified by this approach (Heyer et al. 2012). All sRNAs identified with the experimental RNomics approach were also detected in the HTS analysis. The HTS approach identified also a new class of sRNAs, which were recently discovered in eukaryotes: tRNA-derived fragments (tRFs) (Cole et al. 2009; Lee et al. 2009). Several tRNA 5′ fragments, tRNA 3′ fragments and tRNA 3′ trailer sequences have been identified as stable molecules in eukaryotic cells. In human cells these fragments are generated from mature tRNAs or precursor-tRNAs and represent an abundant class of small RNAs. The tRNA 3′ processing endonuclease tRNase Z (Cole et al. 2009; Hartmann et al. 2009; Lee et al. 2009) as well as Dicer (Jinek and Doudna 2009) seem to be involved in generation of these fragments. Several tRFs have been shown to be associated with Argonaute proteins (Haussecker et al. 2010). Recent studies suggest that these tRNA fragments are not random by-products of tRNA biogenesis and degradation but are an abundant and novel class of short regulatory RNAs, which show specific expression patterns (summarized in Sobala and Hutvagner 2011). Lee et al. (2009) could show that overexpression of a tRF in human cell lines results in higher proliferation rates.

The HTS approach used with *H. volcanii* revealed the presence of 11 tRFs, suggesting that these types of small RNAs are also active in archaea. Northern blot analyses showed the stable presence of these fragments in the cell (Heyer et al. 2012).

The detection of tRFs in *Haloferax* suggests that these are also used in Archaea to regulate gene expression. One potential biological function of these molecules might be the inhibition of translation. In addition, archaeal tRFs might be associated with RNA interacting proteins as seen in eukaryotes. It is apparent that more detailed analyses are required to confirm these speculations and to elucidate their specific role in the cell.

### Functional analysis

After the identification of the pool of potential sRNAs the next and crucial step is to select the best candidates for regulatory RNAs for further in depth studies. One approach to do that is the construction of sRNA gene deletion mutants and the analysis of their phenotypes in comparison to wild type strains. For *H. volcanii* more than ten deletion strains were successfully generated and with two exceptions all were viable. Phenotypic differences between wild type and sRNA gene deletion mutants could be detected under specific conditions such as high temperature, low salt concentrations and different carbon sources (Straub et al. 2009; Fischer et al. 2011; Heyer et al. 2012). For instance, two mutants showed severe growth defects at high temperature and low salt concentrations, respectively (Straub et al. 2009). These data suggest that in *H. volcanii* sRNAs may be required for metabolic regulation.

Very recently, target analysis based on whole transcriptome changes in mutant strains combined with genome-wide in silico target predictions using the tool IntaRNA (Busch et al. 2008) facilitated the identification of the first *trans*-encoded target mRNA for an archaeal sRNA in *M. mazei* (Jäger, Pernitzsch, Backofen, Richter, Sharma and Schmitz, submitted). Demonstration of sRNA sequestering the predicted ribosome binding site as well as the translation start codon of its target mRNA in vitro strongly argues that the interaction is likely to inhibit translation initiation, a mechanisms up to now exclusively described for bacterial sRNAs. Additional evidence was obtained by a genetic approach that this sRNA is involved in regulating the metabolic switch from methanol to methylamines fueled methanogenesis, most likely by the above-mentioned post-transcriptional regulation (Jäger, Pernitzsch, Backofen, Richter, Sharma and Schmitz, submitted).

## Outlook for archaeal sRNA research

The six archaeal organisms studied up until now in regard to their sRNA population represent only the tip of the iceberg for our knowledge about sRNAs in archaea. Furthermore, from the phylum of crenarchaeota only one organism was analyzed and none from the phylum thaumarchaeota. One might also expect a certain diversity of sRNA regulation mechanisms within the archaeal domain. Therefore, much data are required to find out how archaea regulate gene expression with small RNAs.

Nevertheless the data acquired up until now show that archaea encode sRNAs including *trans*-encoded sRNA and *cis*-antisense sRNAs. But the details of the interaction between sRNAs and their targets have not been unraveled yet. Further, it is not known what kind of effect an sRNAs has on its target, whether it, for instance, triggers the degradation of the target mRNA. Also, we do not know yet whether archaeal sRNAs can also act on proteins to regulate gene expression like their bacterial counterparts. In light of the extreme growth conditions found in the archaeal domain the investigation of the sRNA mechanism promises to reveal interesting discoveries about RNA–RNA interactions and RNA–protein interactions. Considering the observation that in some archaea mRNAs contain very short or no 5′ UTRs, an interaction of an sRNA with the 3′ UTR might also be likely. Altogether the analysis of sRNA regulation in archaea will remain fascinating and promises exciting discoveries in the near future.

## CRISPR/Cas, the adaptive immune system of prokaryotes

**Fig. 1:**
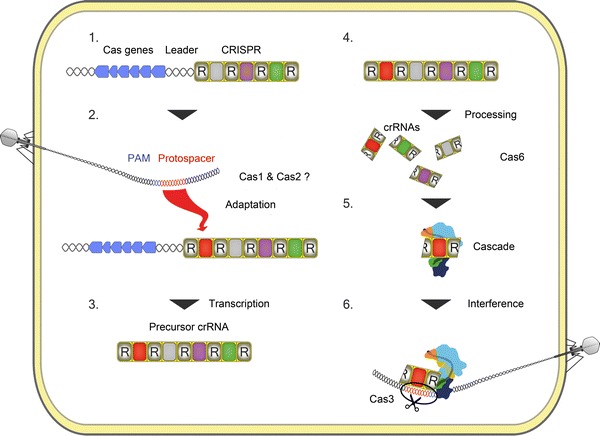
Schematic overview of the CRISPR/Cas type I mechanism. A viral sequence (protospacer, *red*) is incorporated into the host CRISPR cluster (*R* indicates repeat sequences). The universal Cas1 and Cas2 proteins are proposed to mediate this adaptation step. The CRISPR cluster is transcribed into a pre-crRNA that is processed into mature crRNAs by Cas6. The crRNAs are loaded onto the Cascade complex that delivers crRNA to the viral DNA during a repeat attack. The virus is identified via base complementarity between crRNA and protospacer and is subsequently degraded by Cas3 (color figure online)

In the following sections, we would like to shift the focus to a different family of regulatory RNAs, namely the crRNAs that fulfill executive roles in the antiviral defense mediated by CRISPR/Cas systems. CRISPR sequences were initially discovered in *E. coli* in 1987 (Ishino et al. 1987), but only named and classified as a common family of repetitive DNA sequences in the genomes of many bacteria and archaea in 2002 (Jansen et al. 2002). CRISPR is short for clustered regularly interspaced short palindromic repeats, which describes some of the structural features of these clusters (Fig. 1). The nearly identical repeat sequences of a standard CRISPR array vary in length between 23 and 47 nucleotides and are interspaced with so-called spacer sequences of similar size. It was first observed in 2005 that these spacer sequences can represent fragments of the genomes of viruses and other foreign genetic elements (Mojica et al. 2005). Finally in 2007, one major function of the ubiquitous CRISPR arrays was discovered. It was shown that *Streptococcus thermophilus* can acquire resistance against a virus by integrating a genome fragment of this specific virus into its CRISPR cluster (Barrangou et al. 2007). Therefore, CRISPR is described as the adaptive immune system of prokaryotes which contains a set of spacer sequences that dictate specificity. The co-evolution of viruses and their hosts guarantees a constant potential of adding new spacer sequences (and therefore new resistances) to a growing CRISPR locus. Indeed, some CRISPR loci contain several hundred spacer sequences. However, recombination at repeat sequences is a potential mechanism for reducing the total size of repeat-spacer units which is most often found to be averaged between 20 and 30 units. The spacer sequences are inserted at one specific end of the CRISPR loci that is close to a so-called leader region. This leader region was shown to contain a promoter for the transcription of the CRISPR locus (Pul et al. 2010) and proposed to contain elements that direct spacer integration. Several CRISPR-associated (Cas) genes are often found in the direct vicinity of the leader region and the Cas proteins fulfill many critical roles within this prokaryotic immune system which will be detailed below.

**Fig. 2:**
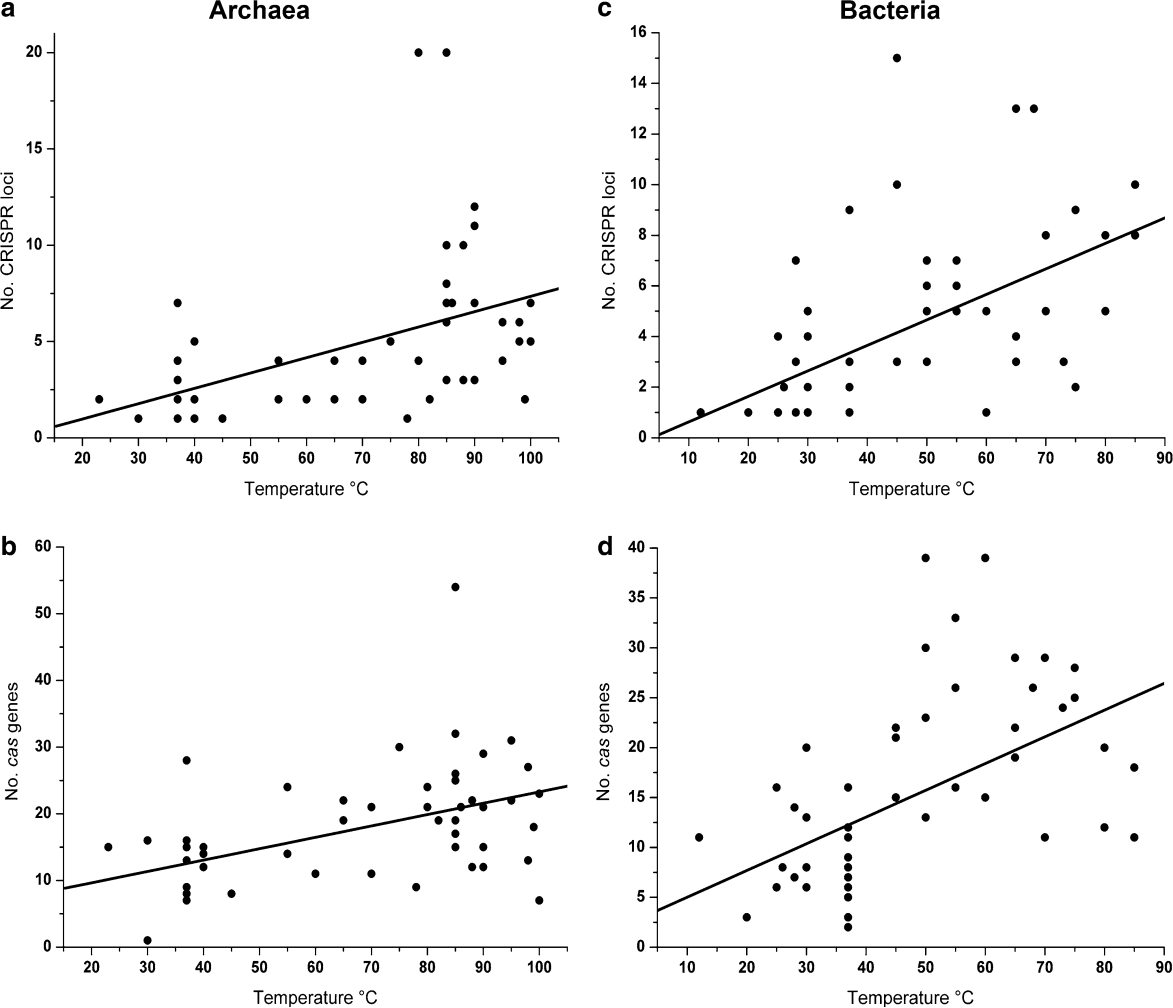
Correlation of archaeal and bacterial growth temperatures with CRISPR/Cas content. Over 50 archaeal (**a**, **b**) and bacterial (**c**, **d**) genomes were analyzed for number of CRISPR and *cas* genes, respectively, and compared to the respective optimal growth temperatures. Indicated is the best-fit line of raw data. Significance of correlation was confirmed by Spearman’s rank correlation test (**a** ρ = 0.459, **b** ρ = 0.602, **c** ρ = 0.609, **d** ρ = 0.602; **a**–**d** *p* < 0.001)

Recent computational studies showcased the divergence of CRISPR/Cas systems in Bacteria and Archaea (Haft et al. 2005; Makarova et al. 2011a, b). CRISPR/Cas types and subtypes differ in length and structure of the repeats but mostly in the set of utilized Cas proteins. CRISPR/Cas systems were classified into three major types and ten subtypes (Makarova et al. 2011a, b). Type I CRISPR/Cas systems are proposed to target only DNA and are distributed in a non-uniform fashion among archaeal and bacterial lineages. Type II CRISPR/Cas systems appear to be found exclusively in Bacteria and utilize a *trans*-encoded guide RNA and host RNase III for crRNA maturation (Deltcheva et al. 2011). Archaea most likely do not possess RNase III-like enzymes (Condon and Putzer 2002) which would explain the observed difference in type II distribution. Type III CRISPR/Cas systems are more commonly found in Archaea and target either RNA or DNA. It is intriguing to see archaeal CRISPR/Cas systems that target RNA even though archaeal RNA viruses have not been described yet. First indications for the existence of archaeal RNA viruses were only recently obtained (Bolduc et al. 2012). It might be that the mRNA presents a target for destruction rather than the DNA genome of an archaeal virus. The analysis of the occurrence of CRISPR clusters within prokaryotic genomes reveals two general trends. First, CRISPR clusters are more often found in archaeal genomes (~90 %) than in bacterial genomes (~40 %) (Grissa et al. 2007) and second, multiple CRISPR clusters are more often occurring in thermophilic organisms than in meso- or psychrophilic strains (Anderson et al. 2011) (Fig. 2). One striking example is the number of CRISPR clusters found within methanogenic archaea. Mesophilic Methanococcus strains have no or very few CRISPR clusters, while some hyperthermophilic Methanocaldococcus genomes contain over 20 clusters that can make up over 1 % of their total genome (Lillestøl et al. 2006).

### Mechanism of CRISPR-mediated immunity

The common principles of CRISPR/Cas functions, the role of individual Cas proteins and the functional differences among the CRISPR subtypes are studied intensively. In several bacteria, *Pyrococcus furiosus* and *Sulfolobus*, some aspects have already been elucidated in substantial detail while other parts remain mostly obscure. The main features of CRISPR-mediated immunity are likely shared between all subtypes and are commonly classified into three main stages that we will follow with a specific viral sequence (in our example spacerX, indicated in red in Fig. 1).

In the first stage, termed adaptation, the viral DNA is injected into a host cell and protospacerX is recognized and targeted for insertion into the host CRISPR. It is not clear how the acquisition of a new spacer is mediated but, based on gene deletion analyses, the universal proteins Cas1 and Cas2 are proposed to play key roles in this process (Brouns et al. 2008). Short conserved sequences (usually 2 or 3 nucleotides) called protospacer adjacent motifs (PAMs) flank the spacer sequence in the viral genome (termed protospacer) and thus determine the targets of most CRISPR/Cas systems (Mojica et al. 2009). CRISPR clusters start and end with a repeat sequence. Therefore, a new repeat has to be generated via an unknown mechanism at the region flanking the leader sequence and spacerX is inserted between this new repeat and the former first repeat. The matrix for the new repeat generation or potential mechanisms for repeat duplication are still to be identified.

The second stage encompasses the transcription of the CRISPR cluster via a promoter sequence within the leader into a long precursor-crRNA (pre-crRNA) which is subsequently processed by Cas6 or related enzymes into short, regulatory crRNAs (Carte et al. 2008; Haurwitz et al. 2010). Cas6 was shown to mature crRNA into a sequence that contains the complete spacer sequence flanked by repeat fragments that define a specific 8-nucleotide 5′-hydroxyl terminus and 2′–3′ cyclic phosphate ends of varying length (Gesner et al. 2011; Jore et al. 2011; Sashital et al. 2011). Thus, one crRNA contains the entire spacerX sequence. Type III CRISPR/Cas systems utilize further Cas proteins for the 3′-terminal maturation of crRNAs after primary processing events (Hatoum-Aslan et al. 2011).

The final stage is termed interference and describes the targeted destruction of viral DNA. A repeated attack by the virus that initially provided the spacer sequence can now be interfered with the use of the now present crRNA containing spacerX. The crRNAs are inserted into a multi-subunit complex called Cascade (CRISPR-associated complex for antiviral defence) (Brouns et al. 2008; Wiedenheft et al. 2011). The Cascade complexes use the crRNA to identify the spacerX sequence in invading viral DNA by base-pairing starting at the 5′ end of the crRNA. The absence of base-pairing between the 5′ and 3′ terminal tags of the crRNA and the viral protospacer and PAM sequence ensure that only viral DNA is cleaved and not host DNA that would otherwise present a target at the CRISPR cluster that generated the crRNA (Marraffini and Sontheimer 2010). The base-pairing between crRNA and protospacer induces conformational changes that might trigger the recruitment of Cas3 in type I CRISPR/Cas systems (Wiedenheft et al. 2011). Cas3 often consists of a helicase domain that mediates the unwinding of RNA/DNA and DNA/DNA duplexes and a HD nuclease domain that finally cleaves the invading single-stranded DNA strand (Beloglazova et al. 2011; Mulepati and Bailey 2011; Sinkunas et al. 2011).

The current general understanding of the impact and activity of CRISPR has been described in detail (Bhaya et al. 2011; Terns and Terns 2011) and we will therefore focus on the description of recent findings for selected archaeal CRISPR/Cas systems.

### CRISPR/Cas systems of Archaea

#### *Pyrococcus furiosus*

Detailed studies of archaeal CRISPR/Cas systems were conducted for *Pyrococcus furiosus* (Hale et al. 2008, 2009). Here, long CRISPR transcripts are processed by Cas6 into crRNA via a single endonucleolytic cut within the repeat that generates the characteristic 5′ terminal 8 nucleotide tags. Cas6 cleavage products are further processed at the 3′ end by a currently unknown mechanism that is thought to involve an exonuclease (Carte et al. 2008). The crystal structure of *P. furiosus* Cas6 is available in complex with a repeat RNA and provides insights into pre-crRNA recognition and cleavage (Wang et al. 2011). Cas6 is a metal-independent endoribonuclease that is proposed to cleave pre-crRNAs via a general acid–base chemistry trans-esterification reaction employing an active-site catalytic triad that is also commonly found in archaeal tRNA splicing endonucleases (Calvin and Li 2008). RNA substrate recognition differs between Cas6 homologs and *P. furiosus* Cas6 was shown to bind to the 5′ terminus of unstructured pre-crRNA substrates, while other Cas6-like homologs (Cse3 and Csy4) recognize a stable hairpin the lies immediately upstream of their cleavage sites (Gesner et al. 2011; Sashital et al. 2011; Wang 2012). Structural studies on the Cas6 protein of *Pyrococcus horikoshii* suggest that Cas6 can form dimers in an RNA sequence-dependent manner, which were proposed to benefit the efficiency and specificity of endonuclease activity (Wang et al. 2012).

Other studies on the *P. furiosus* CRISPR focused on the effector complex of CRISPR/Cas subtype III-B. This complex consists of Cas proteins that are often found in a separate module. The common occurrence of a conserved structural motif termed RAMP (repeat associated mysterious protein) within these proteins coined the classification as Cmr (Cas module RAMP) proteins. Isolated *P. furiosus* Cmr module complexes contained six to seven Cas proteins (Cmr 1–1, Cmr 1–2, Cmr 2–6) and mature crRNAs (Hale et al. 2009, 2012). In contrast to the Cascade complex of *E. coli* and probably other type I and II CRISPR/Cas systems, the Cas RAMP module (CMR) complex targets and cleaves RNA instead of DNA (Hale et al. 2009). Cleavage activity depends on divalent cations and is specific for single-stranded RNA substrates that are complementary to the crRNA that is bound to the CMR complex. The omission of any of the six Cmr proteins but Cmr5 in the effector complex assembly reduced cleavage activity significantly (Hale et al. 2009). Finally, it could be shown that synthetic crRNAs could be designed to direct the targeting and cleavage activity towards an mRNA of choice (Hale et al. 2012). Thus, the Cmr complex can use such crRNAs with a defined 8 nt 5′ tag and a guide sequence that is complementary to a target mRNA to mediate the cleavage of this molecule. These experiments suggest that Cmr complexes have the potential to play a role in the regulation of endogenous mRNAs.

#### *Sulfolobus*

Processed archaeal crRNAs were first detected experimentally in *Archaeoglobus fulgidus*, *Sulfolobus solfataricus* and *Sulfolobus acidocaldarius* as part of RNomics approaches to identify small RNAs (Tang et al. 2002, 2005; Lillestøl et al. 2006). Effector complexes of both a DNA-targeting Cascade-like I-A subtype complex and an RNA-targeting CMR subtype III-B complex have been identified in *Sulfolobus solfataricus* (Lintner et al. 2011; Zhang et al. 2012). The archaeal Cascade-like complex (aCascade) was shown to contain Cas7, Cas5, Csa5, the crRNA maturation enzyme Cas6 and crRNA. Other Cas proteins might be associated in vivo. Nevertheless, recombinant Cas7 and Cas5 proteins are sufficient to form a stable complex that binds crRNAs and complementary ssDNA. The production of recombinant Cas7 yields extended right-handed helical assemblies. These assemblies are thought to support and protect the crRNA along their entire length (Lintner et al. 2011). The model is consistent with the available structures for the *E. coli* Cascade complex (Wiedenheft et al. 2011). However, it is unclear if and how the number of aggregated Cas7 subunits is limited in aCascade as the *E. coli* Cascade complex contains a defined hexameric CasC core.

The CMR complex of *S. solfataricus* consists of seven subunits (Cmr 1–7) and a crRNA (Zhang et al. 2012). Cmr7 is an additional subunit that is not present in the analyzed *P. furiosus* CMR complex and homologs are only identifiable in the *Sulfolobales*. It was demonstrated that the CMR complex cleaves the annealed crRNA and target RNA at UA dinucleotides in the presence of manganese ions. This activity was initially proposed to be mediated by the HD nuclease domain of Cmr2 but recent studies indicated that this component is not the catalytic domain (Cocozaki et al. 2012). One notable difference between the cleavage reactions of CMR complexes from *P. furiosus* and *S. solfataricus* is the generation of different termini structures. *P. furiosus* CMR complexes generate 3′-cyclic phosphate while the *S. solfataricus* CMR complex produces 3′-OH and 5′-phosphate ends.

*S. solfataricus* proves also to be an ideal system to study CRISPR/Cas activity in vivo due to the availability of several viruses and tools for its genetic manipulation (Prangishvili and Garrett 2004; Wagner et al. 2009). An in vivo CRISPR/Cas system is available that follows the targeting of DNA in SSV1 transfection assays (Manica et al. 2011). Furthermore, the strains *S. solfataricus* P2 and *S. islandicus* REY15A were used to follow the changes of the CRISPR loci when challenged with viral and plasmid protospacers on a positively selected plasmid (Gudbergsdottir et al. 2011). These studies revealed that transformation success depended on the deletion of (a) CRISPR loci of different sizes, (b) entire CRISPR/Cas modules but also (c) the precise excision of single spacers that matched the protospacer sequence. The mechanism of this excision event remains to be identified.

#### Other archaea

Since there is only limited sequence information available concerning archaeal viruses it is often not possible to identify PAM sequences for archaeal CRISPR/Cas systems by matching spacer sequences to viral sequences. Therefore, plasmid-based invader sequences have been established and successfully been used (Gudbergsdottir et al. 2011; Fischer, Maier, Stoll, Brendel, Fischer, Pfeiffer, Dyall-Smith, Marchfelder, in revision). A systematic approach using a plasmid-based set-up was used to identify PAM sequences for *Haloferax volcanii* (Fischer, Maier, Stoll, Brendel, Fischer, Pfeiffer, Dyall-Smith, Marchfelder, submitted). Here, a spacer from one of the genomic CRISPR loci was cloned into a *Haloferax* plasmid and potential PAM motifs were added adjacent to this spacer sequence. Using this method, six different PAM sequences were identified which are required for target recognition during the defence reaction in *H. volcanii*. This is the first CRISPR group for which more than two PAM sequences have been identified. Using the plasmid-based invader set-up it was shown that the CRISPR/Cas system of *H. volcanii* is targeting DNA. Cells which could escape the defence reaction did so by deleting the complete *cas* gene cassette or the protospacer on the plasmid or the corresponding spacer in the chromosomal CRISPR locus (Fischer, Maier, Stoll, Brendel, Fischer, Pfeiffer, Dyall-Smith, Marchfelder, in revision).

Analysis of the CRISPR/Cas I-A system of the crenarchaeon *Thermoproteus tenax* revealed two discrete polycistronic transcripts that contained (a) the genes for the aCascade unit (*csa5*, *cas7*, *cas5a*, *cas3*, *cas3*′, *cas8a2*) and (b) a proposed Cascis unit (CRISPR-associated complex for the integration of spacers) (*cas4*, *cas1/2*, *csa1*). The transcription of the *cascade* genes is modulated by changes of environmental parameters, like UV-light exposure or high ionic strength, indicating a tight regulation of *cas* genes in vivo. The two multi-subunit complexes aCascade and Cascis could be generated by the reconstitution of all individually insoluble recombinant proteins encoded within the operons, which suggests interaction and coordination of these Cas proteins (Plagens et al. 2012).

### Archaeal viruses

CRISPR/Cas systems are more often found in Archaea than in Bacteria and extremophilic archaea contain the highest numbers of CRISPR clusters and available spacer sequences (Anderson et al. 2011) (Fig. 1). This is, however, in stark contrast to the very limited available amount of information for potential viruses that could be targeted by the spacer sequences within the crRNAs of extremophilic archaea. The correlation of spacer sequence and matching viral targets is most successful for crenarchaeal acidothermophiles where up to 30 % of the spacer sequences could be assigned to viral or plasmid genomes (Shah et al. 2009). In most other cases, spacer sequences can be used to estimate the number of archaeal viruses and conjugative plasmids that have not been cultured and/or sequenced. With one exception for a single-stranded DNA virus (Pietila et al. 2009), isolated archaeal viruses contain a dsDNA genome and exhibit a fascinating range of virion morphotypes (Prangishvili et al. 2006). Archaeal viruses lyse their host cells less often than bacterial viruses (Bize et al. 2009) and hyperthermophilic viruses tend to integrate into the host genome.

The short spacer sequences directly provide a tiny fragment of a viral genome from a previous infection that can be used to hunt for unknown archaeal viruses in the environment (Snyder et al. 2010). CRISPR spacer sequences were extracted from metagenomic data obtained from acidic hot spring environments in Yellowstone National Park. Viruses were detectable by hybridization of virus-enriched environmental samples to a microarray platform that contained spacer sequence probes (Snyder et al. 2010). The advent of highly sensitive deep-sequencing technologies should enable the sequencing of viral genomes that have been captured by the short CRISPR spacer sequences.

### CRISPR targets: DNA or RNA?

What are the consequences for the CRISPR/Cas system that targets either DNA (e.g. Cascade and Cas3) or RNA (e.g. CMR complex)? The targeting of DNA allows for the usage of spacers that derive from either the sense or the antisense strand of a viral genome. Targeting RNA of mobile genetic elements would limit the choice of protospacers to sequences that are complementary to the given mRNA. On the other hand, DNA targeting poses the problem of discriminating viral DNA from host DNA as any genomic CRISPR sequence would provide a crRNA target. This discrimination is achieved by the use of PAM sequences as described above. PAM sequences were not observed to be required in the targeting of the CMR complex. Therefore, this mechanism might have evolved to free the CRISPR/Cas system from the requirement of certain protospacer flanking sequences and would provide an antiviral defense system that can complement DNA-targeting CRISPR/Cas subtypes.

## Conclusion

The described identification of diverse small non-coding RNA families in archaea showcases the growing realization of the importance of these RNA species in this third domain of life. Modern high-throughput sequencing methods will reveal the RNome composition of further archaeal species. This information can then be combined with necessary functional studies on individual sRNA and crRNA molecules together with the identification of their target sites and should help us to validate the full scope of their influence on cellular processes.

## Abbreviations

sRNA: Small RNA
HTS: High throughput sequencing
crRNA: CRISPR RNA
CRISPR: Clustered regularly interspaced short palindromic repeats
Cas: CRISPR associated
